# Supplementary material for: Heterogeneous nuclear ribonucleoprotein U (HNRNPU) safeguards the developing mouse cortex
Source: Nat Commun. 2022 Jul 21;13:4209. doi: 10.1038/s41467-022-31752-z (PMC9304408; doi:10.1038/s41467-022-31752-z)
Supplement: Supplementary file 1 — Supplementary Information [file 41467_2022_31752_MOESM1_ESM.pdf]

Supplementary Figures

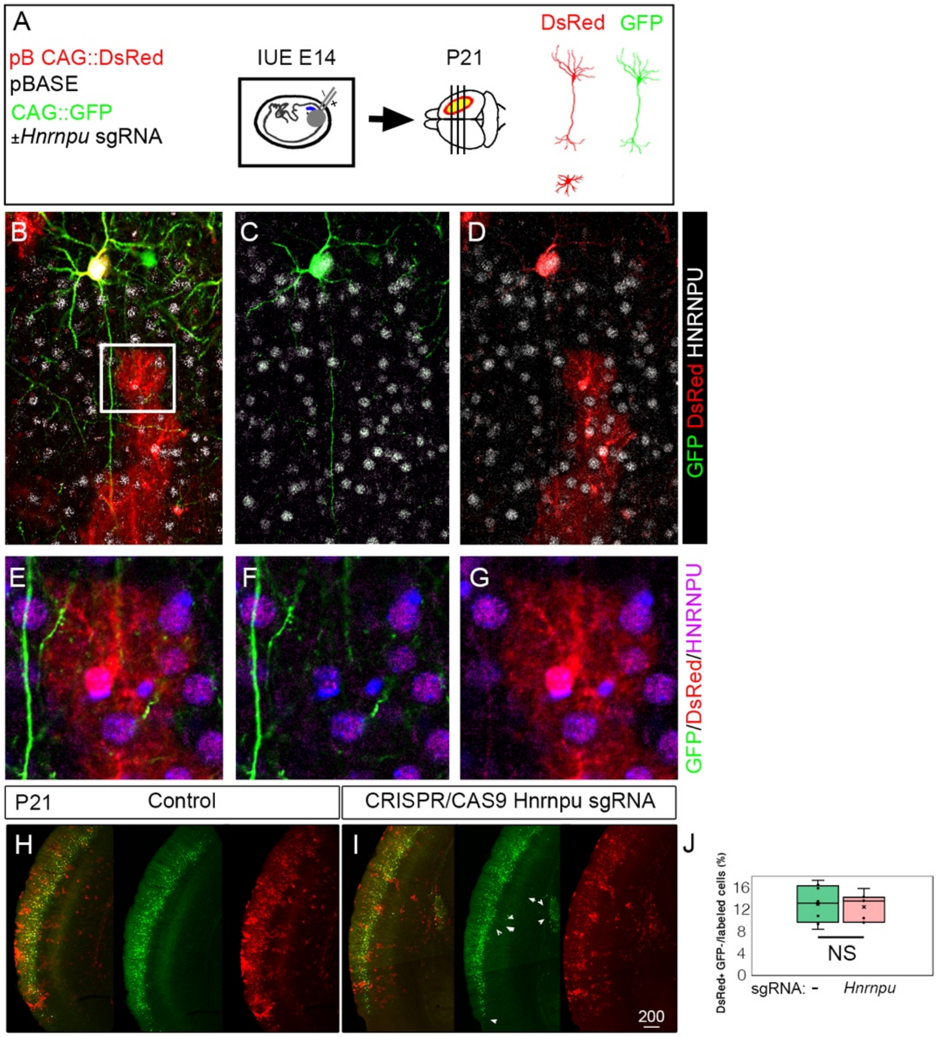

Supplementary Figure 1: Lineage tracing following *in utero* knockdown of *Hnrnpu*.

A) Experimental design. Embryos (E14) were *in utero* electroporated with either control px330 plasmid (-*Hnrnpu* sgRNA) or CRISPR/CAS9 *Hnrnpu* sgRNA (+*Hnrnpu* sgRNA) together with an episomal CAG::GFP and an artificial transposon (pB CAG::DsRed)- transposase (pBASE). B-G) Earlier-born neurons (Red+ GFP+) reside in layer II-IV. Lineage traced astrocytes (Red+ GFP-) express dsRed from the integrated transposon, both express nuclear HNRNPU (white in B-D pink in E-G). The white box indicates the cells shown in higher magnification in E-G. H-J) Treated pups (n=3 of each treatment) were collected at P21 from control (H) or *Hnrnpu* sgRNA (I). Arrowheads indicate ectopic neurons. J) Graphic representation of the proportion of the dsRed+GFP- out of all labeled cells n=8 (Control), n=7 (*Hnrnpu* sgRNA) unpaired t-test. Size bars length is in µM. Error bars (SE) are indicated. Nested test analysis, NS-non significant. Source data are provided as a Source Data file.

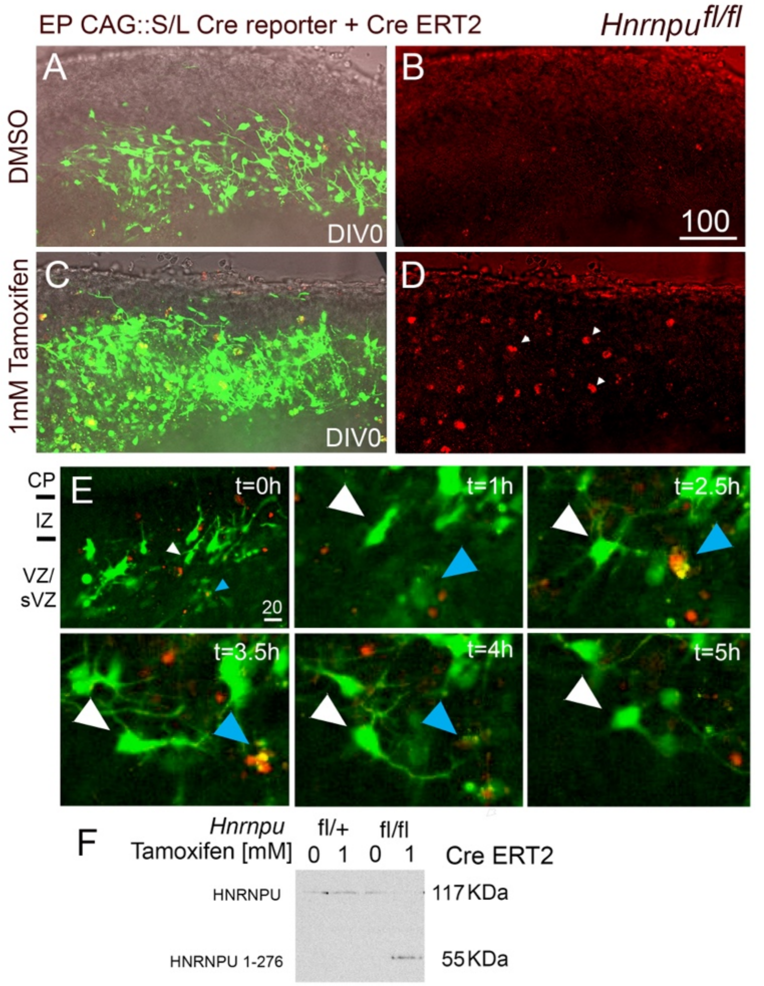

Supplementary Figure 2: <u>*In situ* activation of *Hnrnpu* truncation results in rapid cell death.</u> A-D) Images from a time-lapse movie of live, 300 μM thick organotypic slices of E14 *Hnrnpu*$^{fl/fl}$ brains prepared 24h post *in utero* electroporation. Embryos were electroporated with CAG::Cre ERT2 and CAG Spotlight Cre reporter (CAG::S/L, Green). Tamoxifen (1mM) or solvent (DMSO) were added to the media to induce Cre expression. Successful excision of the reporter is seen (Red). E) Images of five hours long movie from a Tamoxifen treated slice, showing an event of Cre excision (Blue arrowhead) and an adjacent cell (white arrowhead). Accumulation of mCherry is first noticeable at t=1h, and apoptotic death appears at t=2.5h. F) Western blot of tamoxifen-treated neurospheres, prepared from Ub-CreERT2/ *Hnrnpu*$^{fl/fl}$ and *Hnrnpu*$^{fl/+}$ embryos. Truncated HNRNPU (HNRNPU 1-276) is visible in homozygous mice following tamoxifen treatment, using anti-N-ter HNRNPU antibodies.

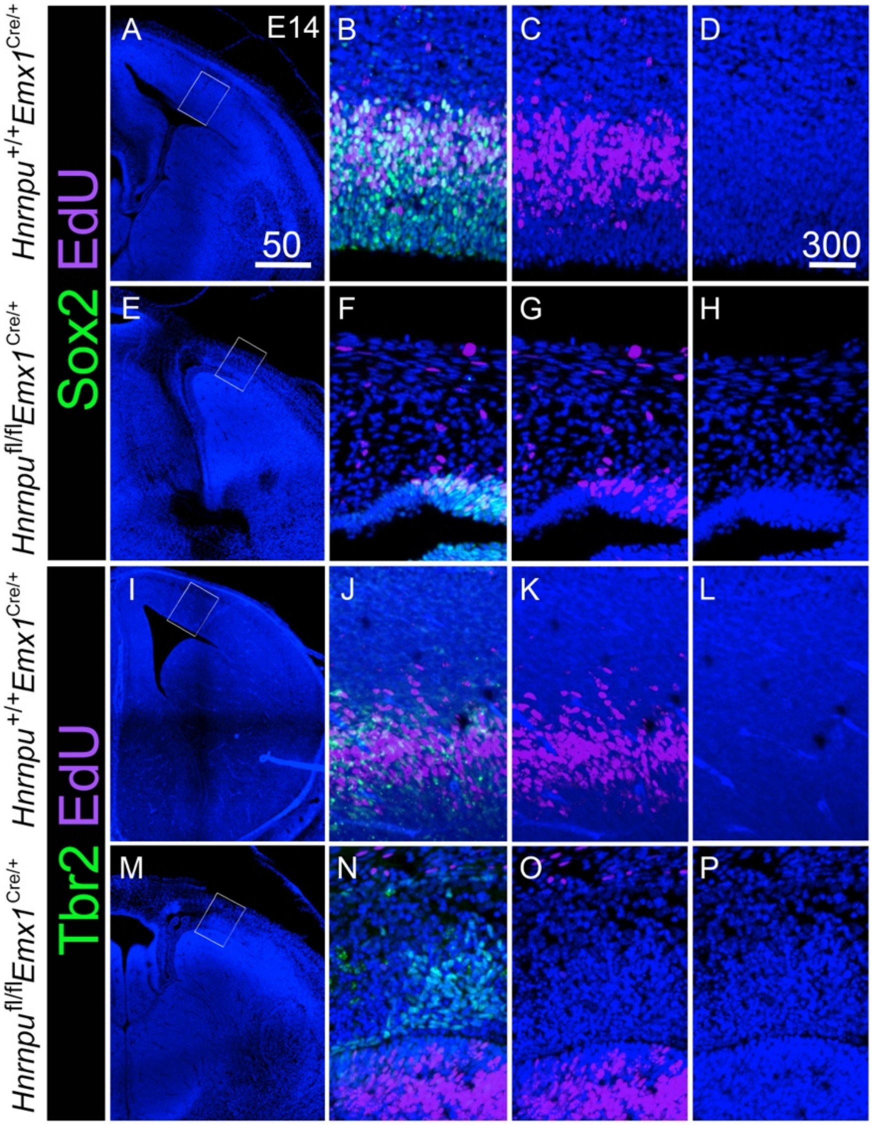

Supplementary Figure 3: Progressive loss of Apical and basal progenitors in *Hnrnpu* deleted embryos. Pregnant mice (E14) were pulse-labeled with EdU (30 min). Brain sections were stained with Sox2 to mark self-renewing neural stem cells and Tbr2 to mark intermediate progenitors. Insert indicate regions immediately adjacent to the extensive cell death at the medial part of the cortex (white boxes). Images reveal reduced VZ/sVZ (expressing Sox2 and Tbr2) and reduced EdU incorporation (F, G vs. B, C and N, O vs. J, K). Size bars are indicated (μM).

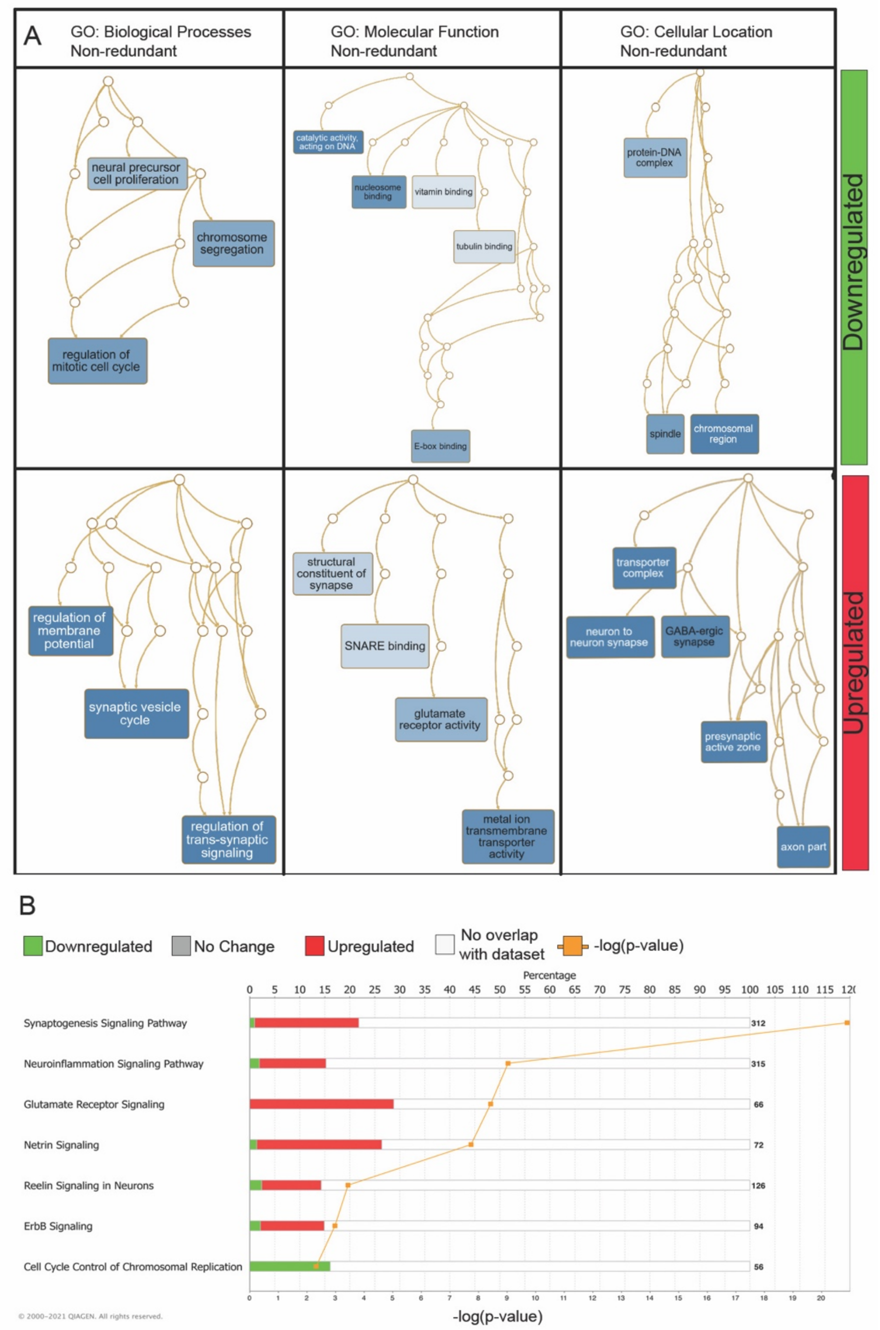

Supplementary Figure 4. A) WebGestalt analysis of DE genes that are upregulated (Red) or downregulated (Green) in the Mutant versus WT belong to different GO terms, displayed as affinity propagation mode. B) Canonical pathway analysis of 1556 differentially expressed (DE) genes between Mutant (*Hnrnpu*$^{fl/fl}$ *Emx1* $^{Cre/+}$) and WT (*Hnrnpu*$^{fl/fl}$ *Emx1*$^{Cre/+}$) was performed with Ingenuity Pathway Analysis IPA (QIAGEN Inc., https://digitalinsights.qiagen.com/IPA) [97].

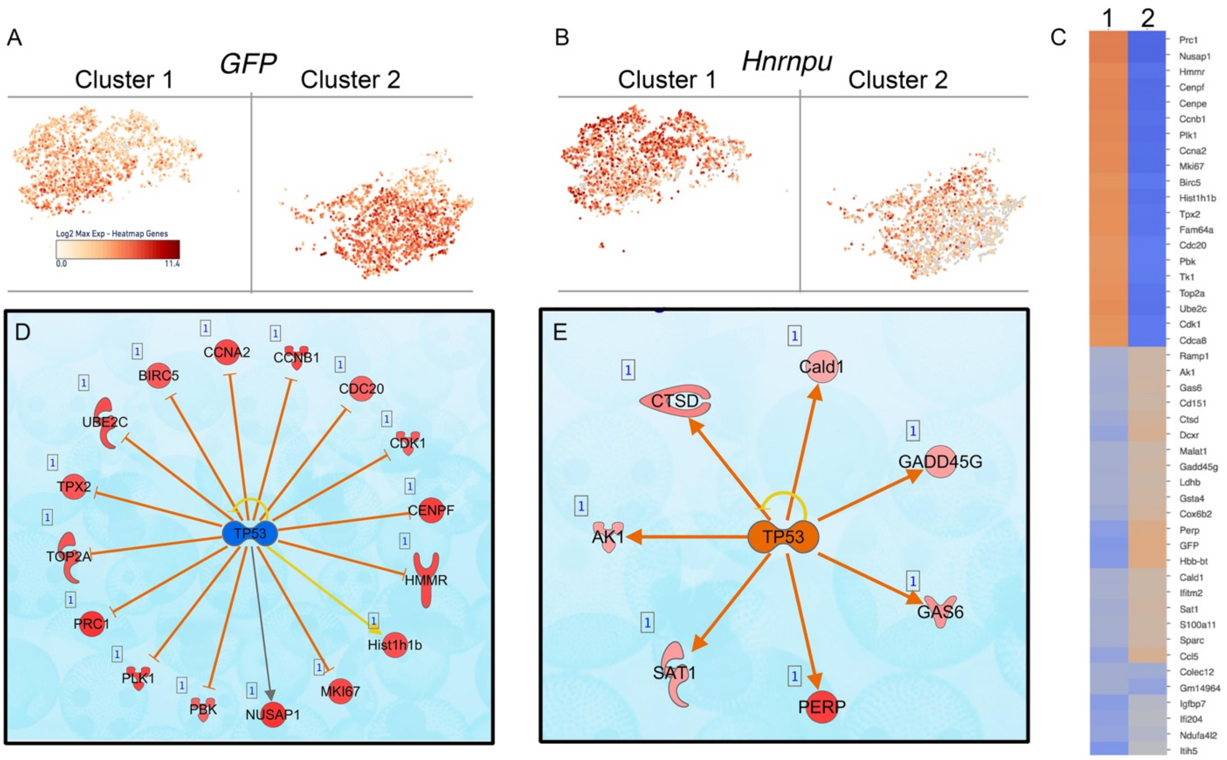

Supplementary Figure 5. Analysis of scRNA-seq data A-C) Co-electroporation of CRISPR/CAS9 *Hnrnpu* sgRNA and GFP expressing plasmid, produce two main complementary clusters (1 and 2 in A, B) based on *GFP* and *Hnrnpu* expression levels. D-E) Upstream pathway analysis of scRNA-seq was performed with QIAGEN Ingenuity Pathway Analysis IPA (QIAGEN Inc., https://digitalinsights.qiagen.com/IPA) [97]. Blue indicates pathway repression; red indicates increased expression; orange indicates pathway activation, and pick increased expression. Orange arrows or blunt arrows indicate that the expression is as expected, grey is unknown, and yellow is inconsistent.

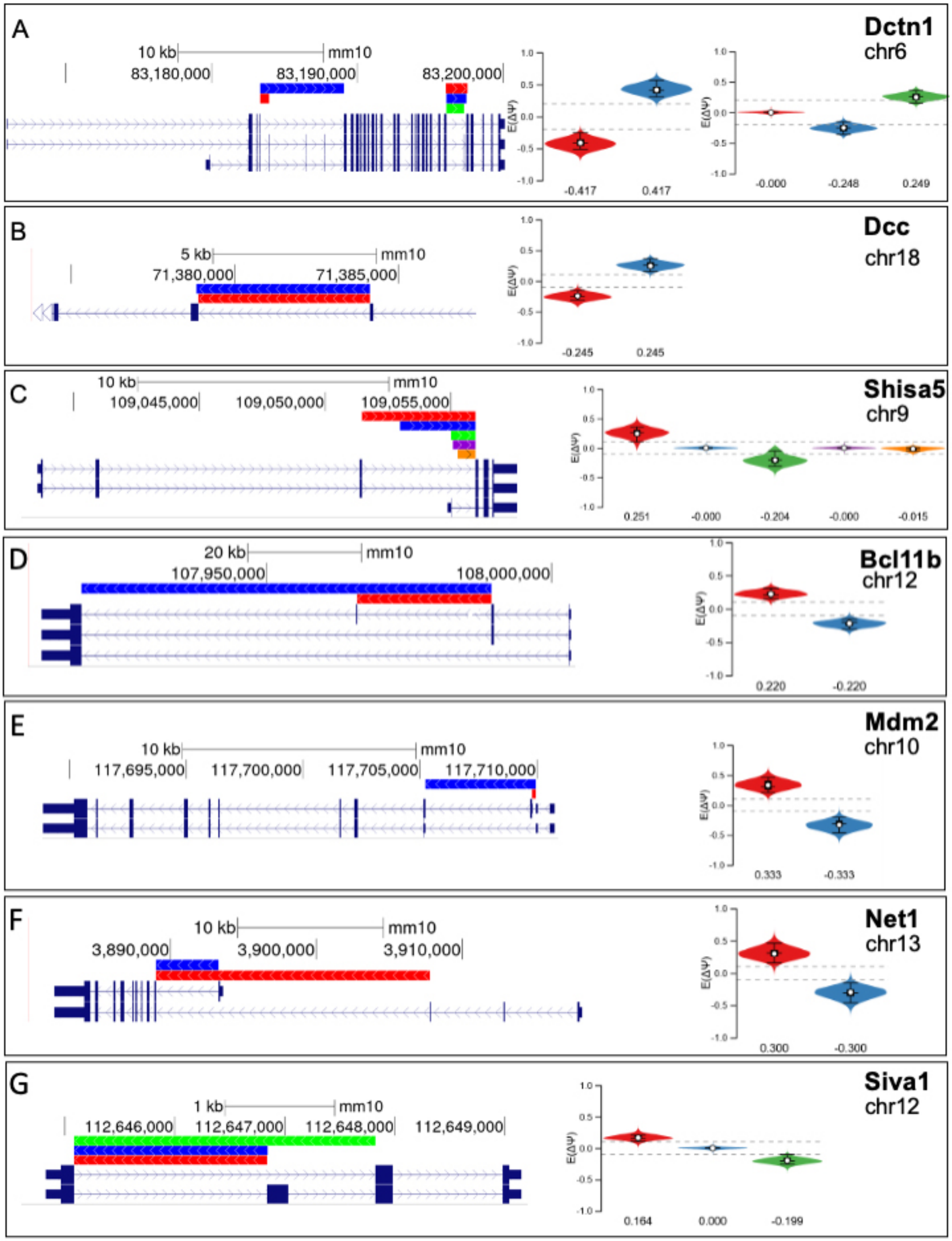
A
Dctn1
chr6
10 kb
mm10
83,180,000
83,190,000
83,200,000
E(ΔΨ)
-0.417
0.417
-0.000
-0.248
0.249
B
Dcc
chr18
5 kb
mm10
71,380,000
71,385,000
-0.245
0.245
C
Shisa5
chr9
10 kb
mm10
109,045,000
109,050,000
109,055,000
0.251
-0.000
-0.204
-0.000
-0.015
D
Bcl11b
chr12
20 kb
mm10
107,950,000
108,000,000
0.220
-0.220
E
Mdm2
chr10
10 kb
mm10
117,695,000
117,700,000
117,705,000
117,710,000
0.333
-0.333
F
Net1
chr13
10 kb
mm10
3,890,000
3,900,000
3,910,000
0.300
-0.300
G
Siva1
chr12
1 kb
mm10
112,646,000
112,647,000
112,648,000
112,649,000
0.164
0.000
-0.199

Supplementary Figure 6: Splicing defects in mouse embryos cortices (E13) following *Hnrnpu* loss of function. Genes with verified differential local splicing variation (LSV) in the Mutant (*Hnrnpu*$^{fl/fl}$ *Emx1* $^{Cre/+}$) vs. WT (*Hnrnpu*$^{fl/fl}$ *Emx1*$^{Cre/+}$). The left side of each panel shows the genomic map of the genes along with the LSV events, highlighted in different colors on top. The right side of each panel shows the difference quantification of each LSV event between the two conditions, measured by the Majiq $\Delta\Psi$ parameter with the same color code as in the genomic map. RNA was collected from n=4 WT and n=4 mutant embryos cortices.

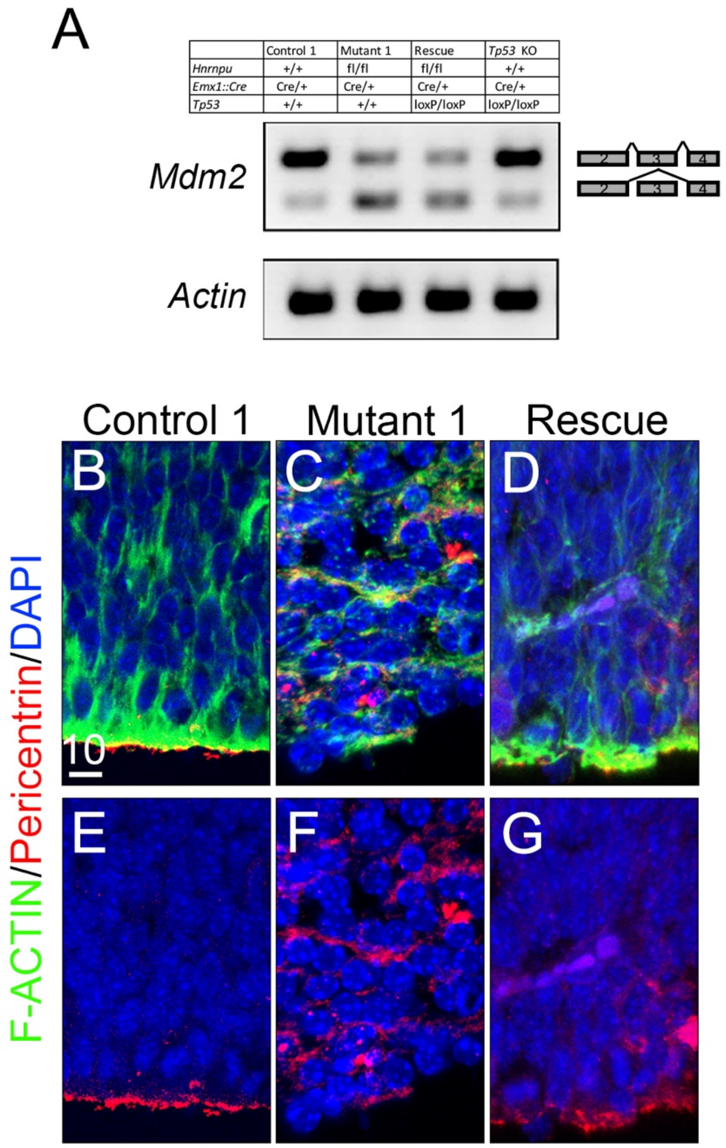

Supplementary Figure 7: A) Rescue of VZ organization in *Hnrnpu* mutant cortices following *Tp53* KO. A) Alternative splicing sensitive PCR detects variants of MDM2, Exon 3 exclusion (300 bp) or inclusion (250 bp) Actin, 150 bp)B-G) F-Actin Belt (Phalloidin 488, Green) and Centrosome tethering (Pericentrin, Red) in E14 WT Cortices (B, E) is lost in mutant littermates (*Hnrnpu*$^{fl/fl}$, *Emx1*$^{Cre/+}$, *Tp53* $^{+/+}$, C, F). Disorganization of VZ architecture is partially restored following Tp53 deletion (*Hnrnpu*$^{fl/fl}$, *Emx1*$^{Cre/+}$, *Tp53* $^{loxP/loxP}$, D, G). Size bar (B), 10μM.

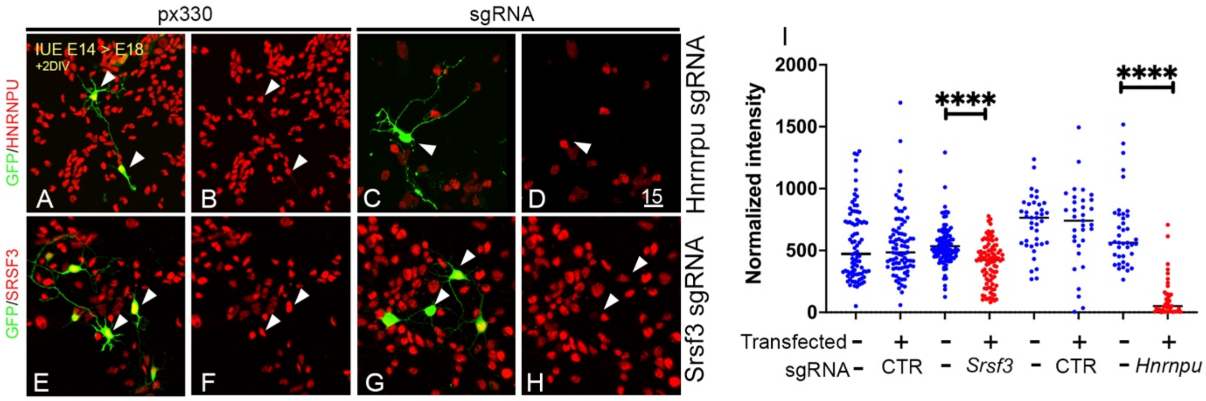

Supplementary Figure 8: *In utero* introduction of CRISPR/CAS9 sgRNA's causes an effective reduction of HNRNPU and SRSF3 protein levels. A-H) Primary cultures (2 days *in vitro*, 2DIV) prepared from E18 brains, four days post electroporation. E14 embryos were coelectroporated with the control plasmid (px330, A-B, E, F), *Hnrnpu* sgRNA (C, D) *Srsf3* sgRNA (G, H) or and were immunostained with anti-N-ter HNRNPU antibodies (A-D) or anti-SRSF3 antibodies (E-F). I) Signal intensity of single cells from the described treatments were normalized against background fluorescence using the Imaris Spot algorithm. Plots show all measured cells (n=39-43 cells of each treatment); horizontal black lines indicate average values. We noticed an average intensity drop of 81.7% in HNRNPU intensity and an average 25.7% drop in SRSF3 intensity following the corresponding sgRNA treatment. ****P<0.0001. Non-significant differences are not indicated.

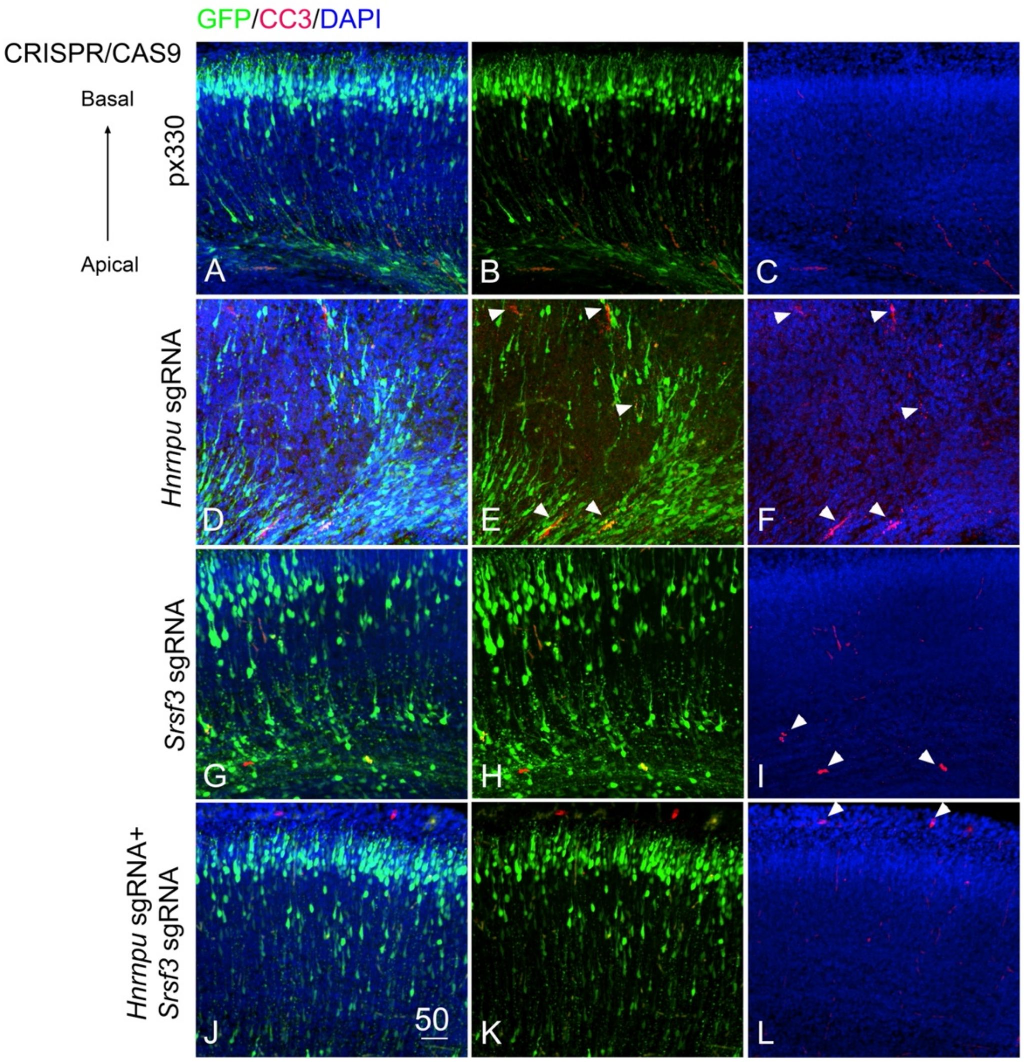

Supplementary Figure 9: Sporadic cell death following E14 *in utero* electroporation with *Hnrnpu* sgRNA. E18 sections from embryos treated with GFP and either px330 (CRISPR/CAS9, no guide, A-C), *Hnrnpu* sgRNA (D-F), *Srsf3* sgRNA (C-I), and a combination of both sgRNA (J-L). Floating sections (70 μM thick) are stained with anti-GFP (Green) to better visualize transfected cells and antibodies to Cleaved Caspase 3 (CC3, Red). Arrowheads indicate cell positive to CC3 staining. Size bar size (J) is 50 μM.

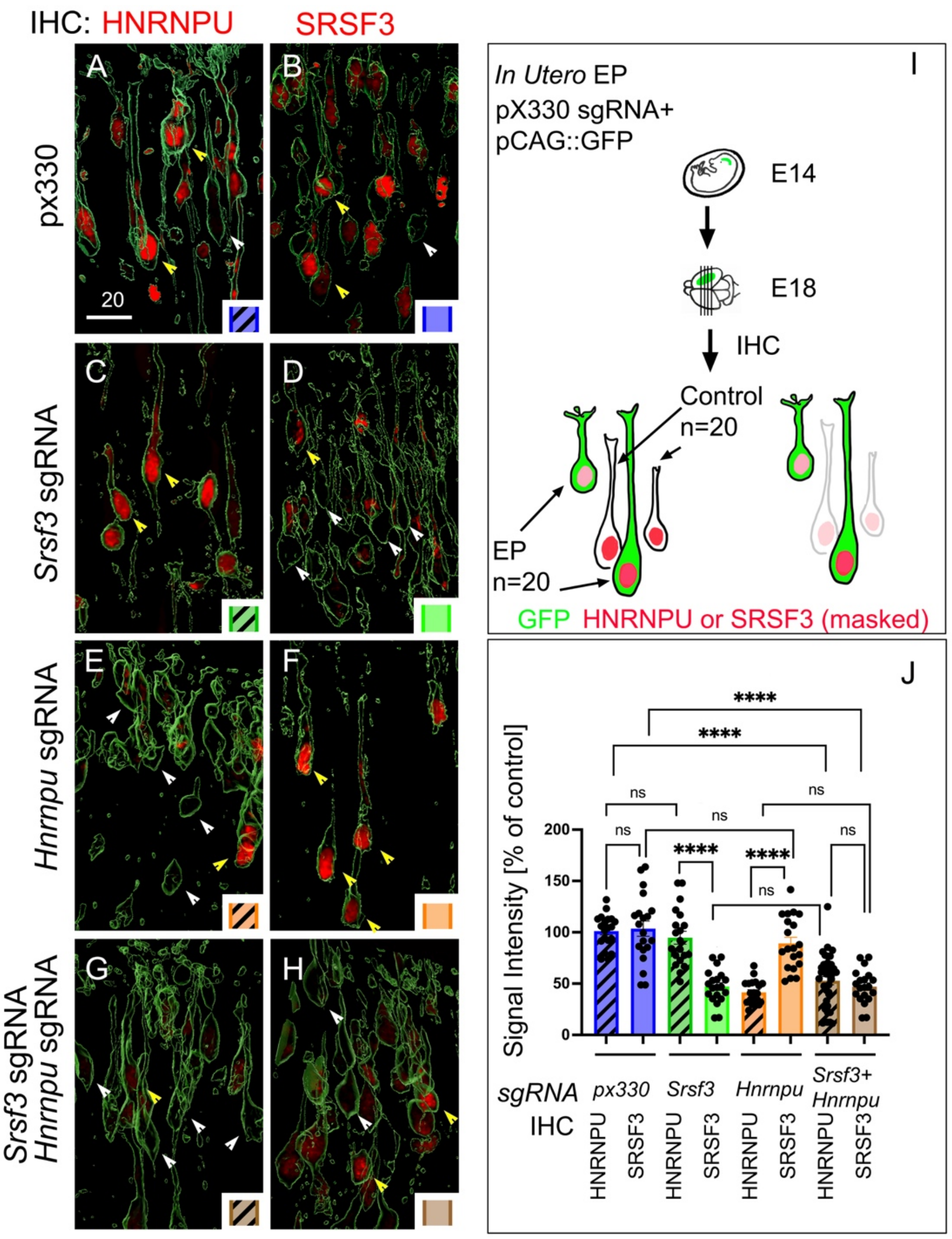

Supplementary Figure 10: <u>Effective reduction of the expression of target proteins.</u> E14 mouse embryos were electroporated with pCAG::GFP and either CRISPR/CAS9 (px330, A-B) *Srsf3* sgRNA (C-D) *Hnrnpu* sgRNA (E-F) or a combination of the two (G-H). Sections were stained with anti- HNRNPU (A, C, E, G, Red) or SRSF3 antibodies (B, D, F, H, Red), and the signal outside the cell volume (Green) was masked. I) Schematic description of the workflow starting with electroporation up to Image analysis. J) Relative intensity of HNRNPU or SRSF3 staining in the electroporated cells (green cells n=20) and non-transfected cells in their vicinity (n=20). In dual sgRNA treatments, n=40 electroporated and n=40 non-transfected cells were measured. Dots represent single measurements. One-way ANOVA. ns- non significant, *** $p < 0.0001$. Error bars (SE) are indicated. Highlighted are random cells in the field with high expression (yellow) or low expression levels (white). The size bar (A) is indicated (20 μM).

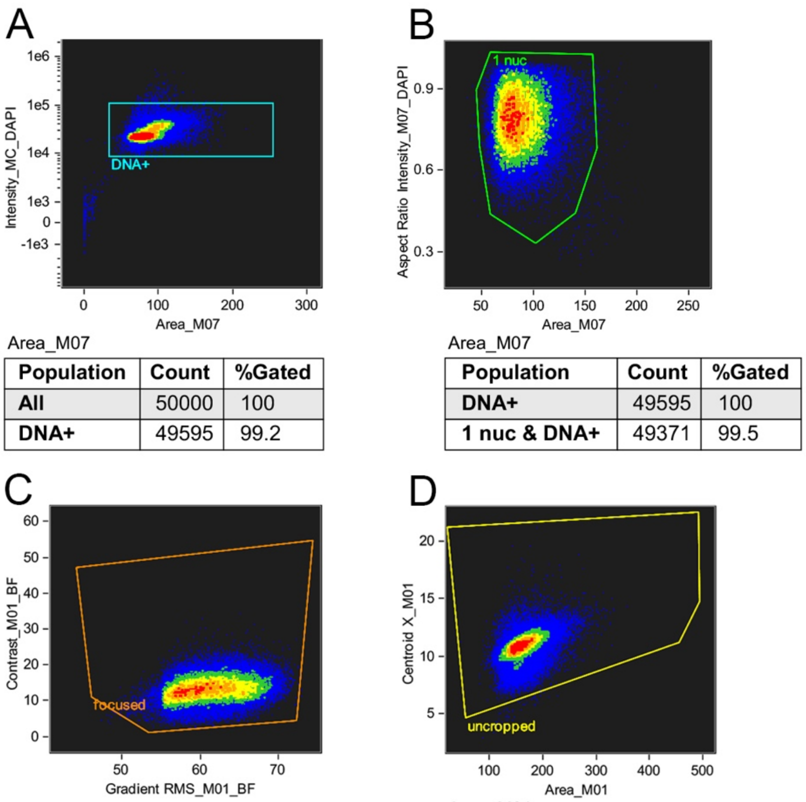

Area_M07

Population	Count	%Gated
All	50000	100
DNA+	49595	99.2

Area_M07

Population	Count	%Gated
DNA+	49595	100
1 nuc & DNA+	49371	99.5

Gradient RMS_M01_BF

Population	Count	%Gated
uncropped & 1 nuc & DNA+	49315	100
focused & uncropped & 1 nuc...	49292	100

Area_M01

Population	Count	%Gated
1 nuc & DNA+	49371	100
uncropped & 1 nuc & DNA+	49315	99.9

Supplementary Figure 11: Gating strategy for imaging flow cytometry.
A-D) At least $5{\times}10^4$ cells were collected from each sample, and data were analyzed using the manufacturer's image analysis software (IDEAS 6.2; Amnis Corp). A) DNA+ cells were gated using the area (the number of microns squared in a mask) and intensity of the DNA staining. B) Single cells were further validated by plotting the area and aspect ratio (the Minor Axis divided by the Major Axis) features on the DAPI channel Low area and high aspect ratio was considered as single cells. C) Focused cells were selected as having high values of Gradient RMS feature and contrast features (measures the sharpness quality of an image by detecting large changes of pixel values in the image) on the bright-field image. D) Cropped cells were further eliminated by plotting the cell area of the bright field image against the Centroid X feature (the number of pixels in the horizontal axis from the left corner of the image to the center of the cell mask).

Supplementary information Table 1: Primary Antibodies

Name	type	Company and catalog number	dilution
Anti-hnRNP U	Up/120<br>rabbit monoclonal	Abcam ab180952	1:200
Anti-hnRNP U	clone 3G6<br>mouse monoclonal	Millipore 05-1516	1:100
Anti-Acetylated Tubulin	clone 6-11B-1<br>mouse monoclonal	Sigma Aldrich T7451	1:500
Anti-CENP-B	C-10<br>mouse monoclonal	Santa Cruz sc-376392	1:50
Anti-p53	CM5<br>rabbit polyclonal	Leica NCL-L-p53-CM5p	1:50
Anti-Cleaved Caspase 3 (Asp 175)	rabbit polyclonal	Cell Signaling 9661	1:100
Anti-phospho-Histone H3 (Ser10)	rabbit polyclonal)	Merck Millipore 06-570	1:200
Anti-Ctip2	rabbit polyclonal)	Abcam ab28448	1:100
Anti-Reelin Antibody	clone G10<br>mouse monoclonal	Sigma-Aldrich MAB5364	1:500
Anti-Tbr1	chicken polyclonal	Merck AB2261	1:100
Anti-Tbr2	chicken polyclonal	Merck AB15894	1:200
Anti-SRSF3/SRP20	rabbit polyclonal	LifeSpan Biosciences LS B11073	1:100
Anti-Pericentrin	mouse<br>monoclonal	BD Bioscience 611814	1:50
Anti-KI67	SP6<br>rabbit polyclonal	Abcam ab16667	1:100
Anti-NeuN	clone A60<br>mouse monoclonal	Merk MAB377	1:500
Anti-Sox2	Rabbit polyclonal	Abcam ab97959	1:300
Anti-GFAP	Rabbit polyclonal	Dako Agilent Z033401-2	1:1000
Anti-CUX1/CDP	M-222<br>rabbit polyclonal	Santa Cruz sc-13024	1:100
Anti-CNPase	D83E10)<br>Rabbit monoclonal	Cell Signaling #5664	1:100

Supplementary information Table 2: Secondary antibodies

Fluorophore	Antibody	Company	Catalog number
Cy™3	AffiniPure Donkey Anti-Rabbit IgG (H+L)	Jackson labs	711-165-152
Cy™3	AffiniPure Donkey Anti-Mouse IgG (H+L)	Jackson labs	715-165-150
Cy™3	AffiniPure Donkey Anti-Chicken IgY (IgG) (H+L)	Jackson labs	703-165-155
DyLight™ 405	AffiniPure Donkey Anti-Rabbit IgG (H+L)	Jackson labs	711-475-152
Alexa Fluor® 488	AffiniPure Donkey Anti-Rabbit IgG (H+L)	Jackson labs	711-545-152
Alexa Fluor® 488	AffiniPure Donkey Anti-Mouse IgG (H+L)	Jackson labs	715-545-150
Alexa Fluor® 488	AffiniPure Donkey Anti-Chicken IgY (IgG) (H+L)	Jackson labs	703-545-155
Alexa Fluor® 647	AffiniPure Donkey Anti-Rabbit IgG (H+L)	Jackson labs	711-605-152
Alexa Fluor® 647	AffiniPure Donkey Anti-Mouse IgG (H+L)	Jackson labs	715-605-150

All secondary antibodies were used in a 1:200 dilution

Supplementary information Table 3: <u>Chemicals</u>

ZVAD-FMK	Sigma Aldrich	V116
Q-VD-OPh Hydrate	Sigma Aldrich	SML0063
Pifithrin-Mu	Sigma Aldrich	P0122
Necrostatin-1	Sigma Aldrich	N9037
(Z)-4-HydroxyTamoxifen	Sigma Aldrich	H7904
EdU (5-ethynyl-2'-deoxyuridine)	Lumiprobe	2426
Fast SYBR Green Master Mix	Thermo Fisher	4385614
Phalloidin Fluorescein Isothiocyanate Labeled	Sigma Aldrich	P5282
Cy3-Azide	Click Chemistry Tools	Az119
Cy5-Azide	Click Chemistry Tools	Az118
ProLong Gold Antifade	Mountant (Invitrogen)	

Supplementary information Table 4: Media

Penicillin Streptomycin solution	Biological Industries	03-031-1B
Matrigel Basement Membrane Matrix	Corning International	354234
Leibovitz L-15 Medium with L-Glutamine	Biological Industries	01-115-1A
Minimum Essential Medium Eagle	Sigma Aldrich	M8042
Neurobasal Medium (1X)	Gibco/Thermo Fisher Scientific	21103-049
B-27™ Supplement (50X)	ThermoFisher	17504044
Gentamicin solution	Sigma	G1272
Animal-Free Recombinant Human EGF	PeproTech	AF-100-15-100
Recombinant Human FGF-basic (154a.a.)	PeproTech	100-18B-100
Heparin Sodium Cell culture tested	Sigma Aldrich	H3149-10KU
Poly-L-Lysine 0.01% Sol.	Sigma Aldrich	P4707
Laminin	Sigma Aldrich	11243217001

Supplementary information Table 5: Kits

EdU-Click 488	Sigma Aldrich	BCK-EDU488
Neural Tissue Dissociation kit (P)	Milteny	130-092-628
RNEASY Mini kit	Quiagen	74104

Supplementary information Table 6: Oligonucleotides

sgRNA mHnrnpu-A	CTTGCCGGCACCACGCTGCTGGG
sgRNA mHnrnpu-B	CTCGCTCTTCGCGGTGACGGTGG
sgRNA mSrsf3-A	CACCGCTCTCAAGTACCTGCGCCG
sgRNA mSrsf3-B	CACCGTAATGACGGATAGATAACC

GENE	Forward	Reverse
Hnrnpu	TGGCAGCAGGGTCAATTCT	CCTTGGTGATAATGCTGACTCC
p53	CACAAAAACAGGTTAAACCCAG	AGCACATAGGAGGCAGAGAC
Cre	AACATGCTTCATCGTCGG	TTCGGATCATCAGCTACACC

Primers

RT-PCR			
RPS29	TCGTTGGGCGTCTGAAGGCAA	CGGAAGCACTGGCGGCACAT	
Arpp21	GACAGCTCTTTCGGGCTCAC	CTTCAGATTGCGGTTGGAACT	PrimerBank: 15149484a1; validated
Cacna1e	GATGGAGACTCGGACCAGAG	TGACCGTGAAACAGTTCTGCC	PrimerBank: 6753230a1; validated
Cacng2	ATGGGGCTGTTTGATCGAGG	GCCAATAGTCGGTTCCCACA	PrimerBank: 6680826a1; validated
Camk2b	GCACGTCATTGGCGAGGAT	ACGGGTCTCTTCGGACTGG	Primer Bank: 291291007c2
Dpp10	AAAGCCTCCAGACACTCACTT	GGGATTCAACTCCCACACTTC	Primer Bank: 238776841c3
Grin2b	GCCATGAACGAGACTGACCC	GCTTCCTGGTCCGTGTCATC	PrimerBank: 6680099a1; validated
Grm5	CCCAGCACAAGTCGGAAATAG	TGTCTGGTTGGGGTTCTCCTT	PrimerBank: 26346991a1; validated
Kcnj10	GTCGGTCGCTAAGGTCTATTACA	GGCCGTCTTTCGTGAGGAC	PrimerBank: 34328498a1; validated
Gria2	TTCTCCTGTTTTATGGGGACTGA	CTACCCGAAATGCACTGTATTCT	PrimerBank: 29387225a1; validated
Kcnq3	GAGCCGACAAAGACGGGAC	CTGTACTTGGCGTTGTTCCTC	Primer Bank: 282398105c1
NeuroD1	ATGACCAAATCATACAGCGAGAG	TCTGCCTCGTGTTCCTCGT	PrimerBank: 33563268a1; validated
Syn1	AGCTCAACAAATCCCAGTCTCT	CGGATGGTCTCAGCTTTCAC	PrimerBank: 18606446a1; validated
Vamp2	GCTGGATGACCGTGCAGAT	GATGGCGCAGATCACTCCC	PrimerBank: 6678551a1; validated
Anxa	ATGTATCCTCGGATGTTGCTGC	TGAGCATTGGTCCTCTTGGTA	PrimerBank: 6754570a1; validated
Apoe	CTGACAGGATGCCTAGCCG	CGCAGGTAATCCCAGAAGC	PrimerBank: 6753102a1; validated
Arpp21	GACAGCTCTTTCGGGCTCAC	CTTCAGATTGCGGTTGGAACT	PrimerBank: 15149484a1; validated
Atf3	GAGGATTTTGCTAACCTGACACC	TTGACGGTAACTGACTCCAGC	PrimerBank: 31542154a1; validated
Bbc3	AGCAGCACTTAGAGTCGCC	CCTGGGTAAGGGGAGGAGT	PrimerBank: 18875398a1; validated
Dcxr	ACTGTGCTGGCGTTGAAGG	CGGGTCCCACATTGCTTAGG	PrimerBank: 13385924a1; validated
Gas6	TGCTGGCTTCCGAGTCTTC	CGGGGTCGTTCTCGAACAC	PrimerBank: 9506715a1; validated
Mdm2	TGTCTGTGTCTACCGAGGGTG	TCCAACGGACTTTAACAACTTCA	PrimerBank: 6754672a1; validated
P21	GGCAGACCAGCCTGACAGAT	TGGGCACTTCAGGGTTTTCT	
Perp	ATCGCCTTCGACATCATCGC	CCCCATGCGTACTCCATGAG	PrimerBank: 11528520a1; validated
Puma	GAGCGGCGGAGACAAGAAG	TCCAGGATCCCTGGGTAAGG	
Sat1	GAGAACACCCCTTCTACCACT	GCCTCTGTAATCACTCATCACGA	PrimerBank: 6677849a1; validated

Semi-quantative PCR			
Actin	GGCTGTATTCCCCTCCATCG	CCAGTTGGTAACAATGCCATGT	PrimerBank: 6671509a1; validated
Dcc-set1	CCTCTCAACCCAAACATCGT	TTTCTGGTTCTTCGGGACAG	
Dcc- set2	TGTTGGCAGTCCTTATGCTG	CAGAAAAGCTGGTCCTCCAC	
Siva1-set1	CGCTCCAACTCAAAGTCCAC	ATACACACTGCCCACACAGG	
Siva1-set2	GCTACTCACGCGAGGTCTTC	CAGCTGGTGCACAGTGTCTT	
Mdm2-set1	GATCACCGCGCTTCTCCTGC	CAATGTGCTGCTGCTTCTCG	
Mdm2-set2	GATCACCGCGCTTCTCCTGC	GATGTGCCAGAGTCTTGCTG	

RT-PCR for alternative splicing			
Shisa5-total	CCCTACCCAACGCAGTACC	GGTCGGGTTGTATGGAGGC	
Shisa5-202-set1	AGGAGACACCCGAACATCTG	AGTTGTGGTGTTGGTCACGA	
Shisa5-202-set2	CGCTGAAATTTCGATCCAGT	GCAGGTGAAGCAGATGATGA	
Shisa5-203-set1	TTGAAAGGGGAGGAGATTGA	AGTTGTGGTGTTGGTCACGA	
Shisa5-203-set2	TTGAAAGGGGAGGAGATTGA	ACAAAGATGGTCACGCCAAT	
Net1-total	GGTCGGAAATGCTAGACATCAA	GTTCCCCTCGGGACAATTCA	PrimerBank: 114145470c2
Net1-201-set1	CGGCGAACGAGAGATGCTC	CTCCTTCAAATCAAGGCTGCTA	PrimerBank: 9790117a1; validated
Net1-201-set2	CCCACACTGGGACTTCACTT	CACCACGAAGGGTAAACGAC	
Net1-203-set1	TCTCCTGCCCATTAAAAGGA	GGTGGACCTGCTGAATGACT	
Net1-203-set2	TCTCCTGCCCATTAAAAGGA	CACCACGAAGGGTAAACGAC	
Dctn1-total-set1	ACCTCTCCTGGAGCAGCAC	CAGGGTCTCCAGCTTCTCCT	
Dctn1-total-set2	CACTGTCCAGGGAAGGAAGT	TCAGGAGTCTCTGGGGAAGT	
Dctn1-total-set3	GGAGACCACGACTGTGTCCT	CGCTCAGGTCAAACTTCTCC	
Dctn1-203-set1	CTTCTGCTTCAAAGGTCCTCA	CCGTCGAGTTGTGGTCTTTC	
Dctn1-203-set2	ACTTCCCCAGAGACTCCTGA	CGGTGCCTTCTTAGGCTTC	
Dctn1-203-set3	CGGGGACTGAAGCCTAAGA	GGGCCAAGGGAGCTACTG	
Bcl11b-total	CTGGGGGACAGCAATCCTTTC	TGTGGGTCCAAGTGATGGC	
Bcl11b-exon2	CCCGACCCTGATCTACTCAC	GGAGGTGGACTGCTCTTGT	
Bcl11b-exon3	CCTCCGTGATTACTTCACCTCT	TGACCCTCACCCTGAGTCC	
Jnk1-total	AGCAGAAGCAAACGTGACAAC	GCTGCACACACTATTCCTTGAG	PrimerBank: 7710060a1
Jnk1-201-set1	TCTTGATTTTGGACTGGCGAG	CCCATGATGCACCCAACTG	
Jnk1-201-set2	GGGAGAAATGATCAAAGGTGGT	TGAATTCAGGACAAGGTGTTCC	
Jnk1-205-set1	TCTTGATTTTGGACTGGCGAG	CCCATAATGCACCCCACAGA	
Jnk1-205-set2	TGGTCTGTGGGGTGCATTAT	GAAAAGCACATCGGGGAACA	
