# Supplementary material for: Heterogeneous nuclear ribonucleoprotein U (HNRNPU) safeguards the developing mouse cortex
Source: Nat Commun. 2022 Jul 21;13:4209. doi: 10.1038/s41467-022-31752-z (PMC9304408; doi:10.1038/s41467-022-31752-z)
Supplement: Supplementary file 3 — Description of Additional Supplementary Files [file 41467_2022_31752_MOESM3_ESM.pdf]

Description of Additional Supplementary Files

File Name: Supplementary Data 1
Description: The list of Differentially Expressed (DE) genes. A list of differentially expressed (DE) genes using total RNA seq. Normalized read counts are shown. The comparisons of heterozygous versus wild type and mutant versus wild type are shown with the indicated log2 fold change, and adjusted p values (padj).

File Name: Supplementary Data 2
Description: Alternatively spliced genes detected by MAJIQ. The first sheet indicates the list of alternatively spliced genes in the homozygous versus wild type comparison. The second sheet shows a list of high confidence alternatively spliced genes, indicating those genes that are also alternatively spliced in the heterozygous versus wild type comparison. The third sheet indicates the list of alternatively spliced genes in the heterozygous versus wild type comparison.

File Name: Supplementary Data 3
Description: The list of DE genes detected by MARSeq. A list of differentially expressed (DE) genes using MARSeq. Normalized read counts are shown. The comparisons of control versus mutant, control versus rescue, mutant versus rescue, are shown with the indicated log2 fold change, and adjusted p values (padj).

File Name: Supplementary Movie 1
Description: Time-lapse recording (5 h long, images taken every 5 min) of primary cultures (E13) prepared from electroporated cortices with Dual Color Cre reporter (CAG::SL). Images were taken 24 hours post plating.

File Name: Supplementary Movie 2
Description: Time-lapse recording (5 h long, images taken every 5 min) of primary cultures (E13) prepared from electroporated cortices with Dual Color Cre reporter (CAG::SL) and CAG::NLS-Cre-GFP. Images were taken 24 hours post plating.

File Name: Supplementary Movie 3
Description: Time-lapse recording (5 h long, images taken every 5 min) of primary cultures (E13) prepared from electroporated cortices with Dual Color Cre reporter (CAG::SL) and Tbr2::Cre. Images were taken 24 hours post plating.

File Name: Supplementary Movie 4
Description: Time-lapse recording (5 h long, images taken every 5 min) of primary cultures (e13) prepared from electroporated cortices with Dual Color Cre reporter (CAG::SL) and Ta1::Cre. Images were taken 24 hours post plating.
