# Supplementary material for: Heterogeneous nuclear ribonucleoprotein U (HNRNPU) safeguards the developing mouse cortex
Source: Nat Commun. 2022 Jul 21;13:4209. doi: 10.1038/s41467-022-31752-z (PMC9304408; doi:10.1038/s41467-022-31752-z)
Supplement: Supplementary file 11 — Reporting Summary [file 41467_2022_31752_MOESM11_ESM.pdf]

nature portfolio

Corresponding author(s): Reiner, Orly

Last updated by author(s): Jun 2, 2022

Reporting Summary

Nature Portfolio wishes to improve the reproducibility of the work that we publish. This form provides structure for consistency and transparency in reporting. For further information on Nature Portfolio policies, see our Editorial Policies and the Editorial Policy Checklist.

Statistics

For all statistical analyses, confirm that the following items are present in the figure legend, table legend, main text, or Methods section.

n/a	Confirmed	
☐	☒	The exact sample size (*n*) for each experimental group/condition, given as a discrete number and unit of measurement
☐	☒	A statement on whether measurements were taken from distinct samples or whether the same sample was measured repeatedly
☐	☒	The statistical test(s) used AND whether they are one- or two-sided<br>*Only common tests should be described solely by name; describe more complex techniques in the Methods section.*
☒	☐	A description of all covariates tested
☐	☒	A description of any assumptions or corrections, such as tests of normality and adjustment for multiple comparisons
☐	☒	A full description of the statistical parameters including central tendency (e.g. means) or other basic estimates (e.g. regression coefficient) AND variation (e.g. standard deviation) or associated estimates of uncertainty (e.g. confidence intervals)
☐	☒	For null hypothesis testing, the test statistic (e.g. *F*, *t*, *r*) with confidence intervals, effect sizes, degrees of freedom and *P* value noted<br>*Give P values as exact values whenever suitable.*
☒	☐	For Bayesian analysis, information on the choice of priors and Markov chain Monte Carlo settings
☒	☐	For hierarchical and complex designs, identification of the appropriate level for tests and full reporting of outcomes
☐	☒	Estimates of effect sizes (e.g. Cohen's *d*, Pearson's *r*), indicating how they were calculated

Our web collection on statistics for biologists contains articles on many of the points above.

Software and code

Policy information about availability of computer code

Data collection	Leica DMi8 and an Andor Dragongfly 202, spinning disc confocal, Fusion Software version 2.3.0.44
Data analysis	No codes developed. Imaris software 9.5.1 (Bitplane, Zurich, Switzerland) (RRID:SCR_007370). GraphPad Prism 9, GraphPad Software,La Jolla California USA, www.graphpad.com

For manuscripts utilizing custom algorithms or software that are central to the research but not yet described in published literature, software must be made available to editors and reviewers. We strongly encourage code deposition in a community repository (e.g. GitHub). See the Nature Portfolio guidelines for submitting code & software for further information.

Data

Policy information about availability of data

All manuscripts must include a data availability statement. This statement should provide the following information, where applicable:

- Accession codes, unique identifiers, or web links for publicly available datasets
- A description of any restrictions on data availability
- For clinical datasets or third party data, please ensure that the statement adheres to our policy

All data generated or analyzed during this study are included in this published article (and its supplementary information files) and are deposited in NCBI (GEO accession GSE181527).

Human research participants

Policy information about studies involving human research participants and Sex and Gender in Research.

Reporting on sex and gender	N/A
Population characteristics	N/A
Recruitment	N/A
Ethics oversight	N/A

Note that full information on the approval of the study protocol must also be provided in the manuscript.

Field-specific reporting

Please select the one below that is the best fit for your research. If you are not sure, read the appropriate sections before making your selection.

☒ Life sciences ☐ Behavioural & social sciences ☐ Ecological, evolutionary & environmental sciences

For a reference copy of the document with all sections, see nature.com/documents/nr-reporting-summary-flat.pdf

Life sciences study design

All studies must disclose on these points even when the disclosure is negative.

Sample size	All sample sizes are indicated. In case of animal studies a minimal number was used to achieve significance.
Data exclusions	No data was excluded
Replication	Biological replicates (n=3-4, as specified in the text) were used in animal and mRNA analyses. Replications were sucessfull and all were included in the analysis. Number of cells, or neurospheres used in this study are indicated in the text.
Randomization	Randomization was applied when primary cells were used but was not applicable for animals, due to the extreme observable phenotype and the low number of rescued animals.
Blinding	The phenotype we observed was very severe, and was easily recognized, therefore binding was not possible.

Reporting for specific materials, systems and methods

We require information from authors about some types of materials, experimental systems and methods used in many studies. Here, indicate whether each material, system or method listed is relevant to your study. If you are not sure if a list item applies to your research, read the appropriate section before selecting a response.

Materials & experimental systems

n/a	Involved in the study	
☐	☒	Antibodies
☒	☐	Eukaryotic cell lines
☒	☐	Palaeontology and archaeology
☐	☒	Animals and other organisms
☒	☐	Clinical data
☒	☐	Dual use research of concern

Methods

n/a	Involved in the study	
☒	☐	ChIP-seq
☒	☐	Flow cytometry
☒	☐	MRI-based neuroimaging

Antibodies

Antibodies used	Supplementary information Table 1: Primary Antibodies<br><br>Anti-hnRNP U

Up/120
rabbit monoclonal
Abcam ab180952
1:200
Anti-hnRNP U
clone 3G6
mouse monoclonal
Millipore 05-1516
1:100
Anti-Acetylated Tubulin
clone 6-11B-1
mouse monoclonal
Sigma Aldrich T7451
1:500
Anti-CENP-B
C-10
mouse monoclonal
Santa Cruz sc-376392
1:50
Anti-p53
CM5
rabbit polyclonal
Leica NCL-L-p53-CM5p
1:50
Anti-Cleaved Caspase 3 (Asp 175)
rabbit polyclonal
Cell Signaling 9661
1:100
Anti-phospho-Histone H3 (Ser10)
rabbit polyclonal)
Merck Millipore 06-570
1:200
Anti-Ctip2
rabbit polyclonal)
Abcam ab28448
1:100
Anti-Reelin Antibody
clone G10
mouse monoclonal
Sigma-Aldrich MAB5364
1:500
Anti-Tbr1
chicken polyclonal
Merck AB2261
1:100
Anti-Tbr2
chicken polyclonal
Merck AB15894
1:200
Anti-SRSF3/SRP20
rabbit polyclonal
LifeSpan Biosciences LS B11073
1:100
Anti-Pericentrin
mouse monoclonal
BD Bioscience 611814
1:50
Anti-KI67
SP6
rabbit polyclonal
Abcam ab16667
1:100
Anti-NeuN
clone A60
mouse monoclonal
Merk MAB377
1:500
Anti-Sox2
Rabbit polyclonal
Abcam ab97959
1:300
Anti-GFAP
Rabbit polyclonal
Dako Agilent Z033401-2
1:1000
Anti-CUX1/CDP

M-222
rabbit polyclonal
Santa Cruz sc-13024
1:100
Anti-CNPase
D83E10)
Rabbit monoclonal
Cell Signaling #5664
1:100

Supplementary information Table 2: Secondary antibodies
Fluorophore
Antibody
Company
Catalog number
Cy™3
AffiniPure Donkey Anti-Rabbit IgG (H+L)
Jackson labs
711-165-152
Cy™3
AffiniPure Donkey Anti-Mouse IgG (H+L)
Jackson labs
715-165-150
Cy™3
AffiniPure Donkey Anti-Chicken IgY (IgG) (H+L)
Jackson labs
703-165-155
DyLight™ 405
AffiniPure Donkey Anti-Rabbit IgG (H+L)
Jackson labs
711-475-152
Alexa Fluor® 488
AffiniPure Donkey Anti-Rabbit IgG (H+L)
Jackson labs
711-545-152
Alexa Fluor® 488
AffiniPure Donkey Anti-Mouse IgG (H+L)
Jackson labs
715-545-150
Alexa Fluor® 488
AffiniPure Donkey Anti-Chicken IgY (IgG) (H+L)
Jackson labs
703-545-155
Alexa Fluor® 647
AffiniPure Donkey Anti-Rabbit IgG (H+L)
Jackson labs
711-605-152
Alexa Fluor® 647
AffiniPure Donkey Anti-Mouse IgG (H+L)
Jackson labs
715-605-150
All secondary antibodies were used in a 1:200 dilution

Validation

The antibodies were verified by the companies (all the antibodies are commercially available).

Animals and other research organisms

Policy information about studies involving animals; ARRIVE guidelines recommended for reporting animal research, and Sex and Gender in Research

Laboratory animals

The strains: conditional Hnrnpu allele received from Prof. Maniatis (Ye et al., 2015). Emx1-Cre B6.129S2-Emx1tm1(cre)Krj/J Stock No:005628, The Jackson Laboratory. Tp53 LoxP/LoxP (Marino et al. 2000). http://genesdev.cshlp.org/content/14/8/994.long. UBC-Cre-ERT2 B6.Cg-Ndor1Tg(UBC-cre/ERT2)1Ejb/1J Stock No: 007001, The Jackson Laboratory Hsd:ICR(CD1) ENVIGO (Harlan). Both sexes were used in the study.
The ages include embryonic ages E13, E14, E18.
Postnatal: p8, p10, p21.

Housing conditions were added to the text.

	The mice were kept in the animal facility at a temperature of 22°C ±1°C, 50% ±10% humidity, and a 12 hour light/dark cycle.
Wild animals	No wild animals were used in this study
Reporting on sex	Both sexes were used in the study.
Field-collected samples	No field collected samples were collected in this study.
Ethics oversight	All animal work included in the manuscript are covered by accepted IACUC protocols. Animal protocols were approved by the Weizmann Institute Institutional Animal Care and Use Committee.

Note that full information on the approval of the study protocol must also be provided in the manuscript.
